# Shiga-toxigenic *Escherichia coli* O157 in Agricultural Fair Livestock, United States

**DOI:** 10.3201/eid1205.050984

**Published:** 2006-05

**Authors:** James E. Keen, Thomas E. Wittum, John R. Dunn, James L. Bono, Lisa M. Durso

**Affiliations:** * United States Department of Agriculture, Clay Center, Nebraska, USA;; † Ohio State University, Columbus, Ohio, USA;; ‡ Louisiana State University, Baton Rouge, Louisiana, USA

**Keywords:** Escherichia coli O157-H7, STEC, prevalence, livestock, research

## Abstract

Agricultural fairs exhibiting livestock are increasingly implicated in human Shiga-toxigenic *Escherichia coli* O157:H7 (STEC O157:H7) outbreaks. To estimate livestock STEC O157:H7 prevalence at US fairs, we collected 2,919 fecal specimens at 29 county fairs in 2 states and at 3 state fairs in 2002. Fly pools were also collected. STEC O157:H7 was isolated from livestock at 31 (96.9%) of 32 fairs, including 11.4% of 1,407 cattle, 1.2% of 1,102 swine, 3.6% of 364 sheep and goats, and 5.2% of 154 fly pools. Cattle, swine, and flies at some fairs shared indistinguishable STEC O157:H7 isolate subtypes. In 2003, a total of 689 ambient environmental samples were collected at 20 fairgrounds 10–11 months after 2002 livestock sampling while fairgrounds were livestock-free. Four beef barn environmental samples at 3 fairgrounds yielded STEC O157:H7. These data suggest that STEC O157 is common and transmissible among livestock displayed at agricultural fairs and persists in the environment after the fair.

## Abstract 2

Organisms were common in ruminants, swine, and pest flies.

Each year, ≈3,500 state and county fairs in the United States attract >125 million urban, suburban, and rural visitors (*1*). Livestock exhibits, which are popular and common at most of these fairs, provide an opportunity for both direct and indirect human contact with animals that may be subclinically infected with zoonotic enteric pathogens.

Fair attendance increases infection risk for human Shiga-toxigenic *Escherichia coli* O157:H7 (STEC O157) in the United States (*2*). Since 1999, at least 7 US human STEC O157 outbreaks have been associated with visits to agricultural fairs displaying livestock, resulting in thousands of illnesses, >300 culture-confirmed infections, at least 36 cases of hemolytic uremic syndrome, and 2 deaths (*1**,**3**,**4*). Fair STEC O157 outbreaks in the United States have been associated with ruminant contact, contaminated water, and contact with animal environments (*2**,**5**,**6*).

The objectives of this study were to estimate fecal STEC O157:H7 prevalence in livestock on display at US agricultural fairs and to estimate STEC O157:H7 prevalence in the postfair environment, 10–11 months later, when animals were absent. Because pest flies may act as vectors of STEC O157:H7 (*7*) and are abundant at fairs, we also estimated STEC O157:H7 prevalence in flies at fairs. Finally, we compared clonality and estimated diversity of STEC O157:H7 isolates from animals and flies at fairs and from the postfair environment.

## Methods

### Fecal and Fly Sampling

We collected fresh fecal specimens at 32 agricultural fairs at 29 small or local county fairs in 2 midwestern states and at 3 large state fairs in 2 midwestern states and 1 southern state. County fair fecal sampling targeted 25 cattle and 25 pigs. State fair fecal sampling targeted 60–70 each for market and breeding beef, market and breeding swine, and dairy cattle. Other livestock fecal specimens (e.g., sheep, goats, equids, and poultry) were collected as available. To maximize the likely number of source farms per fair from which samples originated, we obtained 1 fecal specimen per cow or 1 fecal specimen per pen for animals displayed in small groups (pigs, sheep, goats, and poultry) with a common owner. If present, adult muscoid pest flies (house flies, *Musca domestica*; stable flies, *Stomoxys calcitrans*; and blow flies, *Calliphoridae* sp.) were trapped live with fly pheromone-baited jug traps or live-netted from livestock buildings, dumpsters, garbage cans, feed containers, and animal wash stations. Fecal and fly samples were collected in summer and early fall of 2002 while fairs were open to the public. Permission to collect samples at fairs was obtained in advance from fair boards or fair managers and, in some cases, from individual animal owners. Fair, animal owner, and animal anonymity were maintained as a condition of permission to sample.

### Environmental Sampling

We collected fairground environment samples from 19 county fairgrounds in 2 states and 1 state fairground in the summer of 2003 from among the 32 fairs visited for livestock sampling in 2002. At the time of environmental sampling, none of the fairgrounds had any livestock; the 19 county fairgrounds (but not the state fairground) had had no or very limited livestock on the premises since the fair in the previous year. Environmental sampling in 2003 was conducted 10–11 months after the 2002 livestock sampling at each fair. Samples collected included soil, bedding (sand, sawdust, woodchips), pest flies, dried manure, standing water, and surface swabs of concrete, wood, and metal structures such as floors, walls, and railings.

County fairground environmental sampling consisted of >30 samples per fairground, 10 each from cattle, swine, and show arena areas. Of each set of 10, a total of 5 were collected at ground level, and 5 were collected from above-ground surfaces. For the state fairground, 25 samples were collected each from the cattle, swine, and show arena areas. Of each set of 25, a total of 10 were from the ground level, 10 were from above-ground surfaces, and 5 were from ceilings.

### Isolation, Serotyping, and Characterization of STEC O157

Fecal enrichment was performed as previously described in gram-negative broth containing vancomycin (8 mg/L), cefixime (0.05 mg/L), and cefsulodin (10 mg/L) (GN-VCC) for 6 h at 37°C, followed by immunomagnetic separation (IMS) (*8**–**10*). Bead IMS aliquots were spread plated onto ChromAgar O157 (CHROMagar, Paris, France) containing 0.63 mg/L potassium tellurite (1× tellurite ChromAgar O157 [TCA]), except for IMS beads derived from enriched swine feces, which were plated onto ChromAgar O157 containing twice (1.25 mg/L) the potassium tellurite (2× TCA). Live-trapped, adult pest fly pools were chilled at –20°C until immobile but viable, counted, speciated, placed into sterile Whirl-Pak bags (Nasco, Fort Atkinson, WI, USA), and crushed with a solid glass rod. Fly broth was enriched by adding either 2 mL of 1.5× (60 g/L) brilliant green bile broth (BGB) or GN-VCC per 5 flies added directly to the Whirl-Pak bags in which the flies were crushed. The bags were incubated for 6 h at 37°C, analyzed by IMS, and plated on 1× TCA. Environmental samples were enriched in 1.5× BGB for 6 h at 37°C, analyzed by IMS, and plated on 1× TCA (*4*). Up to 5 mauve-pink STEC O157 colonies per 1× or 2× TCA plate were serotyped by enzyme immunoassay using monoclonal antibodies to *E*. *coli* O157 and *E*. *coli* H7 (*8*) and analyzed by polymerase chain reaction (PCR) assays for *stx*1, *stx*2 (Shiga toxin), *eae* (intimin), *rfb*_O157_ (O157 O-antigen), and *fliC*_H7_ (H7 flagellum) genes (*11**,**12*). Depending on the O:H antigens and gene subsets present, individual fecal, fly, or environmental isolates identified as *E*. *coli* O157 were classified as STEC O157:H7, Shiga-toxin gene PCR-negative (*stx*-negative) *E*. *coli* O157:H7, or *stx*-negative *E*. *coli* O157:non-H7.

### Pulsed-Field Gel Electrophoresis and Strain Diversity

We conducted pulsed-field gel electrophoresis (PFGE) on representative subsets of fecal, fly, and environmental STEC O157:H7 isolates by using the PulseNet protocol and the restriction endonuclease *Xba*I (*13*). After determining the number and uniqueness of each PFGE pattern for isolates at each fair, we calculated the Simpson diversity index (D) as a measure of within-fair isolate diversity (*14**,**15*). The Simpson D ranges from 0 to 1; higher values represent greater strain diversity. In this case, the Simpson D was the probability that any 2 randomly selected isolates from a given fair had a different (unique) PFGE pattern.

## Results

### Prevalence of *E*. *coli* O157 in Fair Animal Feces and Pest Flies

A total of 2,919 livestock fecal samples were collected at 32 fairs, of which 187 (6.4%) were STEC O157:H7 positive. Species-specific STEC O157:H7, *stx*-negative *E*. *coli* O157:H7, and *stx*-negative *E*. *coli* O157:non-H7 fecal isolation rates are shown in Table 1. STEC O157:H7 was most prevalent in cattle feces (11.4% of 1,407 beef and dairy cattle). Fair-specific sampling intensities and *E*. *coli* O157 isolation rates for cattle and swine are shown in the Figure. STEC O157:H7 prevalence at fairs was 0%–36% in cattle and 0%–8% in swine. We commonly isolated *stx*-negative *E*. *coli* O157:H7 and *stx*-negative *E*. *coli* O157:non-H7 from cattle and swine, as shown in the Figure. Flies live-trapped at 21 fairs generated 154 fly pools (63 stable fly, 54 house fly, and 37 blow fly). STEC O157:H7 was isolated from 8 (5.2%) pools (7 house fly and 1 blow fly) at 4 fairs. STEC O157:H7-positive fly pools originated from beef barns (6 pools), a swine barn, and an outdoor manure pile. We isolated STEC O157:H7 from 7 of 87 fly pools enriched in 1.5× BGB and 1 of 67 fly pools enriched in GN-VCC.

**Table 1:** *Escherichia coli* O157 livestock fecal or pest fly isolation rates from 32 US county and state fairs, 2002*

Sample	No. samples collected	No. (%) STEC O157:H7 positive	No. (%) *stx*-negative *E. coli* O157:H7 positive	No. (%) *stx*-negative *E. coli* O157:non-H7 positive
Beef cattle	1,163	151 (13.0)	9 (0.8)	7 (0.6)
Dairy cattle	244	10 (4.1)	0	11 (4.5)
Pigs	1,102	13 (1.2)	9 (0.8)	19 (1.7)
Sheep	251	11 (4.4)	0	6 (2.4)
Goats	113	2 (1.8)	0	1 (0.9)
Other livestock†	46	0	0	0
Fly pools	154	8 (5.2)	1 (0.7)	7 (4.6)
All samples	3,073	195 (6.3)	19 (0.6)	51 (1.7)
All livestock	2,919	187 (6.4)	18 (0.6)	44 (1.5)

*STEC, Shiga-toxigenic *Escherichia coli*. †Includes 15 chickens, 14 rabbits, 10 horses, 2 alpacas, 1 llama, 1 donkey, 1 pony, 1 turkey, and 1 guinea fowl sampled at 8 county fairs.

**Figure:**
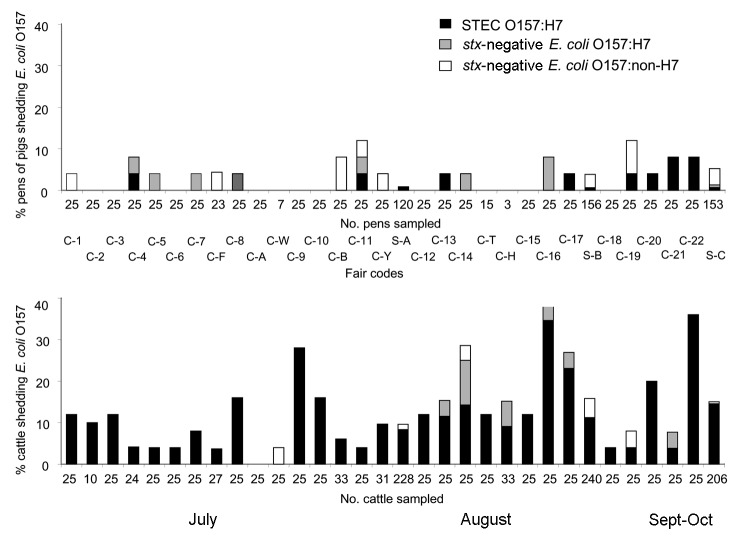
Fecal prevalence of Shiga-toxigenic (*stx*) *Escherichia coli* (STEC) O157:H7, *stx*-negative *E*. *coli* O157:H7, and *stx*-negative *E*. *coli* O157:non-H7 in 1,102 pens of pigs and 1,407 cattle (244 dairy cattle and 1,163 beef cattle) during exhibitions at 3 US state fairs and 29 county fairs, 2002. C, county; S, state. Data are presented in the order that fairs were sampled.

STEC O157:H7 was isolated from >1 livestock species at 28 of 29 county fairs and all 3 state fairs (fair-level prevalence 96.9%). Cattle and swine feces were collected at all fairs, while other livestock were variably present for sampling. Flies were unavailable for sampling at 11 fairs primarily because of inclement weather. The fair-level STEC O157:H7 prevalence by species (i.e., number of fairs with STEC O157:H7 present in the species/number of fairs with this species present) was beef cattle, 30/32 (93.8%); dairy cattle, 4/5 (80.0%); pigs, 11/32 (34.4%); sheep, 6/12 (50.0%); goats, 1/5 (20.0%); other livestock, 0/8 (0%); and pest flies, 4/21 (19.0%).

*E*. *coli* O157 negative for *stx* was prevalent in fair livestock and flies and was found at 19 of the 32 fairs. *E*. *coli* O157:H7 negative for *stx* was isolated from 19 samples at 13 fairs (9 beef cattle, 9 pigs, and 1 fly pool). *E*. *coli* O157:non-H7 negative for *stx* was found in 51 samples at 12 fairs (7 beef cattle, 11 dairy cattle, 19 pigs, 6 sheep, 1 goat, and 7 fly pools).

### Prevalence of Postfair Environmental *E*. *coli* O157

Of 689 environmental samples collected at 20 fairgrounds in the summer of 2003, STEC O157:H7 was isolated from 4 (0.6%) samples at 3 fairgrounds (1 state fairground and 2 county fairgrounds, 15% of sampled fairgrounds). All 4 STEC O157:H7–positive samples were from beef barn environments: 2 dirt samples, 1 house fly pool, and 1 above-ground surface swab. One *stx*-negative *E*. *coli* O157:H7 and 3 *stx*-negative *E*. *coli* O157:non-H7 were isolated from beef barn samples at 2 county fairgrounds.

### Isolate Characterization and PFGE Patterns

Shiga-toxin and intimin gene profiles of 214 livestock and pest fly *E*. *coli* O157:H7 isolates are shown in Table 2. Most of these isolates (90.7%) had *stx*2 alone or in combination with *stx*1. Among the 195 (187 livestock feces and 8 pest fly) STEC O157:H7 isolates, all were *eae*-positive except for 1 pig isolate. Among the 4 environmental STEC O157:H7 isolates, 3 were *stx*1 positive and 1 was *stx*-1, *stx*-2 positive; all 4 were *eae*-positive.

**Table 2:** Gene profiles of *Escherichia coli* O157:H7 from livestock fecal and pest fly samples collected at 32 state and county fairs by source species, 2002*

Sample	No. samples collected	No. (%) *E. coli* O157:H7-positive samples	Gene profile, no. isolates (%)				
*stx*1 only	*stx*2 only	Both *stx*1 and *stx*2	Neither *stx*1 nor *stx*2	*eae*-positive
Beef cattle	1,163	160 (13.8)	0	99 (61.9)	52 (32.5)	9 (5.6)	158 (98.8)†
Dairy cattle	244	10 (4.1)	1 (10.0)	8 (80.0)	1 (10.0)	0	10 (100)
Pigs	1,102	22 (2.0)	0	6 (27.3)‡	7 (31.8)	9 (40.9)	15 (68.2)§
Sheep	251	11 (4.4)	0	5 (45.5)	6 (54.6)	0	11 (100)
Goats	113	2 (1.9)	0	2 (100.0)	0	0	2 (100.0)
Other livestock	46	0	NA	NA	NA	NA	NA
Fly pools	154	9 (5.8)	0	6 (66.7)	2 (22.2)	1 (11.1)	9 (100.0)
All sources	3,073	214 (6.9)	1 (0.5)	126 (58.9)	68 (31.8)	19 (8.9)	205 (95.8)

*Identified by polymerase chain reaction. *stx*, Shiga toxin; *eae*, intimin; NA, not available. †Both beef cattle *eae*-negative isolates were also *stx*-negative. ‡Includes 1 *eae*-negative swine isolate. §Includes all 13 *stx*-positive swine isolates and 2 *stx*-negative isolates.

PFGE was conducted on a subset of 79 fecal, fly, and environmental STEC O157:H7 isolates, including all isolates derived from 1 state fair and 6 county fairs. PFGE results and Simpson D for each fair's isolates are summarized in Table 3. Diverse PFGE patterns were present at most fairs. We found 47 unique PFGE patterns among the 79 tested isolates from 7 fairs. In 2 instances, STEC O157:H7 PFGE patterns were indistinguishable in bovine and fly isolates from the same fair. In another instance, indistinguishable PFGE patterns were found in cattle, swine, and fly isolates at the same fair. Fly STEC O157:H7 isolates from 1 fair had multiple PFGE patterns. At the 3 fairs where we obtained postfair environmental STEC O157:H7 isolates, no environmental isolate PFGE patterns matched any fecal or fly isolates.

**Table 3:** PFGE patterns (n = 47) of 79 STEC O157:H7 from animal fecal, fly, and environmental samples at 7 selected state and county fairs, 2002 (livestock and fly isolates) and 2003 (environmental isolates)*

Fair code	Total isolates	PFGE patterns	Unique X*ba*I patterns (P1–P47) (sample type† - no. isolates)	Simpson D (95% CI)‡
SF2	35	25	P1 (F-5, B-2), P2 (B-2), P3 (B-2), P4 (B-2), P5 (B-2), P6–P25§	0.96 (0.92–0.99)
CF19	4	2	P26 (B-1, P-1), P27 (E-2)	0.67 (0.49–0.85)
CF20	9	5	P28 (P-1, B-1, F-1), P29 (F-3), P30 (D-1),¶ P31 (B-1), P32 (D-1)	0.83 (0.73–0.94)
CF22	14	6	P33 (B-5, P-2), P34 (S-3), P30 (D-1),¶ P35 (B-1), P36 (B-1), P37 (B-1)	0.74 (0.59–0.89)
CFB	4	4	P38 (Ef-1), P39 (B-1), P40 (B-1), P41 (S-1)	1.00 (NA)
CFY	4	4	P42 (B-1), P43 (B-1), P44 (B-1), P45 (S-1),	1.00 (NA)
CF16	9	2	P46 (B-8),# P47 (B-1)	0.22 (0.00–0.57)

*PFGE, pulsed-field gel electrophoresis; STEC, Shiga-toxigenic *Escherichia coli*. †F, fly (pooled fly sample collected when livestock were present on fair grounds); B, beef cattle; P, pig; E, environment; D, dairy cattle; S, sheep; Ef, environment fly (pooled fly sample collected when fair grounds were closed to the public and no animals were present). ‡Probability that any 2 randomly selected isolates from a given fair have unique PFGE patterns. CI, confidence interval; NA, not available. §Twenty isolates from SF2 each with a unique PFGE pattern from the following samples: 16 beef cattle, 2 sheep, 1 pig, and 1 dairy cow. ¶Isolates from dairy samples at CF20 and CF22 were indistinguishable; fairs were geographically close to each other. #A Stool STEC O157 isolate from a human clinical case-patient (hemorrhagic colitis) who had visited this fair also had this PFGE pattern.

## Discussion

The primary aim of the study was to estimate the fecal STEC O157:H7 prevalence in livestock at agricultural fairs across multiple species, multiple fairs, and multiple states to better understand and manage the zoonotic risk. Our data indicate that STEC O157:H7 is endemic and common in ruminant livestock, especially cattle, exhibited at fairs. In addition, swine and pest flies, particularly house flies, may also be infected by or contaminated with STEC O157:H7, albeit at lower prevalence than in cattle. The STEC O157:H7 fecal prevalence of 13% in beef cattle at US fairs in this study (Table 1) is comparable to the STEC O157:H7 fecal prevalence of 13% in summer feedlot cattle (*16*) but less than the STEC O157:H7 fecal prevalence of 28% in feedlot cattle presented for summer slaughter at midwestern meat processing plants (*9*). The STEC O157:H7 fecal prevalence in swine at fairs in this study (1.2%) is similar to the STEC O157 prevalence of 2.0% reported for 350 US swine colon samples collected at slaughter (*17*). We isolated STEC O157 from flies at 19.1% of the 21 fairs from which they were trapped. Data on fly STEC O157 prevalence in livestock settings are limited, but STEC O157 has been isolated from adult house flies on cattle farms (*18**,**19*). Although comparing fair STEC O157:H7 prevalence across states was not a study objective, we found no difference in either cattle or swine fecal prevalence at fairs in the 3 states surveyed (data not shown).

Study of STEC O157:H7 prevalence at agricultural fairs to some degree represents a "natural experiment" opportunity to investigate the role that animal and environmental hygiene may play in the epidemiology and control of this zoonotic agent in livestock, i.e., to evaluate the "hygiene hypothesis." Husbandry and management practices for US fair animals differ a great deal from those used for commercial livestock. Animal density is high, and exposure to feces is constant in most US commercial settings where livestock are managed for meat, milk, or fiber production. In contrast, animals raised for show competitions at fairs are typically reared individually or in small groups and are meticulously groomed and individually fed (*20**,**21*). Fair livestock are thoroughly washed and cleaned at least daily for several weeks before and especially during fairs. Exhibitors of livestock are typically diligent about maintaining clean, manure-free stalls because hygiene is a judged outcome in competitions. We hypothesized that this emphasis on animal and environmental hygiene would result in a lower STEC O157 prevalence in fair animals compared with commercially reared livestock. Our data, however, indicate that STEC O157:H7 fecal prevalence in fair animals is similar to that of their commercial counterparts. This finding suggests that preharvest efforts to limit STEC O157:H7 prevalence or control transmission in production livestock based solely on cleaning of animals or their environment are unlikely to be successful.

Our finding that fair livestock STEC O157:H7 prevalence was high and comparable to that in production livestock is important for agriculture and public health officials, fair managers, and fair visitors to consider. In contrast to livestock production settings, where only small numbers of people and few children have animal access and contact, livestock exhibits at fairs attract millions of persons, many of them children. Monitored and controlled human-livestock interaction and contact at fairs is sometimes encouraged. For example, we isolated STEC O157:H7 from feces of a demonstration milk cow at 1 surveyed state fair. Children were encouraged to milk this cow by hand, so this dairy cow had direct contact with hundreds of children each day. Cattle that are fecal-shedding STEC O157:H7 may have concomitant hide contamination at multiple locations and may also shed the pathogen orally (*10*). Many persons likely had contact with this animal's hide. However, to our knowledge, no human STEC O157:H7 infections were associated with this dairy cow. In contrast, a person with hemorrhagic colitis who visited 1 surveyed county fair was infected with an STEC O157:H7 clone that was indistinguishable from multiple fecal isolates found in cattle at that fair (Table 3).

We isolated *E*. *coli* O157 that did not carry Shiga-toxin genes at several fairs from cattle, pigs, sheep, goats, flies, and the fairground environment (Tables 1 and 2, Figure). This finding underscores the importance of thorough characterization of *E*. *coli* O157 isolates because not all *E*. *coli* O157:H7 are STEC O157:H7 and not all *E*. *coli* O157 have the H7 flagellum antigen or corresponding *flic*_H7_ gene. The potential of these isolates to cause human disease is unclear. However, nontoxigenic *E*. *coli* O157:H7/H-negative strains have been associated with sporadic cases and outbreaks of human disease, including hemolytic uremic syndrome, in Europe (*22**,**23*). In addition, *E*. *coli* O157 Shiga-toxin loss during human infection and during in vitro cultivation are documented (*24**,**25*).

Diverse STEC O157:H7 PFGE subtypes were present at most fairs. This finding is not unexpected because fairs represent a temporary (3–14 days) assemblage of animals from many source farms, STEC O157 is endemic in US livestock (*9*), and livestock STEC O157 clones are diverse between source farms (*26*). Individual animals were not tracked in this study. Therefore, when indistinguishable STEC O157:H7 PGFE subtypes occurred at the same fairs, we could not tell if matches were linked to animals from the same farm, if the same subtype occurred simultaneously on 2 geographically isolated farms, or if the clone was transmitted between animals at the fair. The fact that we observed 2 fairs with indistinguishable fly-livestock isolate PFGE patterns suggests that flies, especially house flies, may be local transmission vectors of STEC O157:H7. Kobayashi et al. (*7*) found STEC O157 in adult house fly intestines and showed experimental shedding by flies for up to 3 days postchallenge. Alternatively, flies and animals may have acquired STEC O157 from a common agricultural fair environmental reservoir such as feed, water, or manure. However, a role for pest flies in the transmission of enteric pathogens to humans is plausible (*27*).

STEC O157:H7 was recovered from the 3 animal-free fairground environments. Environmental STEC O157:H7 may represent residual contamination from previous fairs or other animal events. This environmental contamination may be both an animal biosecurity and a zoonotic risk as a potential source of infection to arriving animals or visiting persons, respectively, at future fair events. PFGE analysis of animal isolates from 2002 and environmental isolates from the same fairs in 2003 did not show any shared STEC O157:H7 isolate subtypes. However, considering the broad observed diversity of STEC O157:H7 clones isolated from fair animals and the limited number of samples that we tested by PFGE, the absence of matching PFGE patterns might be expected.

STEC O157:H7 is a substantial public health risk at fairs. STEC O157:H7 is a frequent infecting or contaminating zoonotic pathogen of animals displayed at agricultural fairs and, to a lesser degree, at the animal-free postfair agricultural environment. Given the high prevalence of STEC O157:H7 at fairs, high fecal prevalence in individual cows, many thousands of exhibited livestock at thousands of agricultural fairs, and millions of human visitors to fairs each year, fair-associated human STEC O157:H7 outbreaks might be expected to be more frequent. Fortunately, however, STEC O157:H7 zoonotic transmission from livestock to humans, at least in a clinically overt manner, is relatively rare. Our PFGE data showing that cattle and pigs (that were exhibited in different buildings at these fairs) shared indistinguishable STEC O157:H7 subtypes are compatible with intrafair transmission among livestock. Possible vehicles include STEC O157:H7-contaminated pest flies or fomite (e.g., feed, water, shared equipment) exposures. Similarly, humans may also be cross-infected with STEC O157:H7 by livestock or flies at fairs. Recently published guidelines on human interactions with livestock in public settings (*28**,**29*) provide a baseline for developing strategies to lower the zoonotic risk for human STEC O157:H7 infection at agricultural fairs.
